# Reproduction in life and death: should cancer patients with a poor prognosis be offered fertility preservation interventions?

**DOI:** 10.1530/RAF-23-0047

**Published:** 2023-11-20

**Authors:** Georgina L Jones, Anne-Mairead Folan, Bob Phillips, Richard A Anderson, Jonathan Ives

**Affiliations:** 1 Department of Psychology, School of Humanities and Social Sciences, Leeds Beckett University, Leeds, UK; 2 Hull-York Medical School and Centre for Reviews and Dissemination, University of York, York, UK; 3 Paediatric Oncology, Leeds Children’s Hospital, Leeds, UK; 4 MRC Centre for Reproductive Health, University of Edinburgh, Edinburgh, UK; 5 Centre for Ethics in Medicine, University of Bristol, Bristol, UK

**Keywords:** fertility preservation, poor prognosis, cancer, posthumous, ethics, reproduction

## Abstract

In the context of a cancer diagnosis, fertility preservation interventions are used to mitigate the potential impact of gonadotoxic cancer treatment upon fertility. They provide patients with cancer the option to freeze their reproductive material to have their own biological child following treatment. The evidence suggests some clinicians are less likely to have fertility preservation discussions with patients who have an aggressive or metastatic cancer which has a poor prognosis. Although this is contrary to current policy recommendations, there is a lack of guidance relating to offering fertility preservation in the context of a poor prognosis to support clinicians. Controversy surrounds posthumous reproduction, and whether the wishes of the cancer patient, when living and deceased should take precedence over others’ well-being. We consider the question of whether cancer patients with a poor prognosis should be offered fertility preservation from an ethics perspective. We structure the paper around key arguments to which multiple ethical principles might pertain, first establishing a central argument in favour of offering fertility preservation based on respect for autonomy, before exploring counterarguments. We conclude by proposing that a defeasible assumption should be adopted in favour of offering fertility preservation to all cancer patients who might benefit from it. It is important to recognise that patients could benefit from fertility preservation in many ways, and these are not limited to having a parenting experience. The burden of proof rests on the clinician in collaboration with their multidisciplinary team, to show that there are good grounds for withholding the offer.

**Lay summary:**

When a person is diagnosed with cancer, they may wish to consider undergoing fertility preservation procedures. These procedures give patients a chance to have their own biological child after completing cancer treatment. However, research suggests that cancer patients who have a poor prognosis are less likely to be offered fertility preservation treatment. In this paper, we consider the ethical implications of offering (or not) fertility preservation to this patient group, including using their sperm or eggs to reproduce after their death. We conclude that fertility preservation treatments should be offered to all cancer patients who might benefit from it, and we outline the many ways that benefit from this treatment can be gained. The decision to withhold the offer of fertility preservation treatment should be made between the patient’s clinician and their wider care team. They must be able to provide good reasons to explain why it was withheld.

## Introduction

Cancer treatments (e.g. chemotherapy, radiotherapy and some surgery) can result in loss of fertility – denying cancer patients the opportunity to have their own biological child in the future. Loss of fertility does not affect all patients treated for cancer (ESHRE 2020), but predicting who will be affected is challenging because cancer treatments variably affect reproductive function depending on the patient’s age, cancer diagnosis and specific treatment regimen.

Procedures are now available which, if undertaken before cancer treatment, may preserve fertility (such as egg, embryo, sperm and ovarian/testicular tissue cryopreservation). Given that loss of fertility is often reported by cancer patients to be one of the most distressing side effects of cancer treatment (Peate *et al.* 2009), fertility preservation (FP) options can provide much needed hope at a time when they are coping with significant uncertainty.

Crucially, the value of FP procedures lies in their ability to preserve the opportunity to have, and to then parent, genetically related children, and the best chances of success for these procedures are when they are offered prior to commencing cancer treatment. Consequently, at cancer diagnosis, international guidelines recommend that cancer teams should discuss the impact of cancer treatment on future fertility with the patient (Loren *et al.* 2013, National Institute of Health and Care Excellence 2017, Yasmin *et al.* 2018, ESHRE 2020, Lambertini *et al.* 2020). Ensuring that patients are supported to make the right FP decision for them is essential, because patients surviving cancer will live with the consequences of the choices made about their fertility for the rest of their lives, so it is vital that they are supported to make the right FP decision for them.

Some cancer patients will have a poor prognosis, perhaps the result of a cancer which responds poorly to treatment, or one with widespread metastases to other parts of the body, and there is little chance of cure. In these circumstances, the requirement to discuss FP options seems less clear, as patients in this position are very unlikely to be able to use their stored material to have and raise children.

Instead, it may be possible to use posthumous assisted reproduction (PAR), whereby cryopreserved eggs, embryos or sperm are used after the death of an individual for the purpose of expanding a family (Lawson *et al.* 2016). The notion of posthumous reproduction was first proposed by Mantegazza in 1866, who first discovered that sperm could be frozen and suggested that women whose husbands may have died during a war could benefit from this discovery (Elliot 2004).

In these circumstances, where posthumous reproduction is the only likely option, we consider the question of whether an FP discussion should take place and whether cancer patients with a very poor prognosis should be offered fertility preservation.

Current available guidance for clinical teams is unclear on this question. While there appears to be a consensus that cancer teams should discuss the impact of cancer treatment on future fertility with the patient at diagnosis, some professional and regulatory bodies such as the American Society of Clinical Oncology (Lee *et al.* 2006) and the American Society for Reproductive Medicine (ASRM) (2005) state that this should also apply to those with a poor prognosis, and discussion should take place irrespective of prognosis in female cancer patients of reproductive age (Loren *et al.* 2013, Peccatori *et al.* 2013, Lambertini *et al.* 2016, Munoz *et al.* 2016). Others such as the NICE Clinical guideline [CG156] in the UK and ESHRE recommendations (2020) do not. However, the NICE guideline does state that when deciding to offer FP to people diagnosed with cancer, cancer teams should take into account factors including diagnosis and prognosis, and the viability of stored or post-thawed material. This distinction might reflect differences between publicly vs self-funded health systems rather than different ethical commitments, but it, nonetheless, provides evidence of varied positions and practices that warrant ethical scrutiny.

To provide that ethical scrutiny, we first consider what the purpose of FP is, which we then use as the backdrop against which to consider various arguments for or against offering FP to late stage or prognostically poor cancer patients.

## What is the purpose and process of fertility preservation?

It is important, at the outset, to consider what the purpose of FP treatment in the context of a cancer diagnosis is, because this allows us to clearly articulate the goods that purportedly flow from it. While our aim is not to undertake a simple consequentialist analysis that balances goods against harms, it is, nonetheless, important to have a clear sense of how people benefit from FP and why it is valuable to them.

It seems that there are two kinds of goods that can flow from FP. The first is the good of being able to reproduce genetically, and the second is the ability to be a parent to those genetically related children. The former is independent of the latter, by which we mean it is possible to reproduce genetically without having the experience of parenting the resulting children. In contrast, the latter is dependent on the former. One cannot have the good of parenting genetically related children without first reproducing genetically.

We contend that the purpose of FP, in the context of cancer treatment, is to allow people to experience the second good. The benefit derived from offering FP to patients undergoing cancer treatment is that it leaves open a future where they could be a parent to their genetic offspring. This seems clear for two reasons. First, if we were not concerned with preserving the opportunity to parent genetic offspring specifically, we would not be concerned about fertility preservation at all. We would simply reassure patients that notwithstanding the likely loss of fertility, they will have the opportunity to parent non-genetically related children through gamete donation or adoption. Second, although it is possible to simply preserve reproductive material so that it might be used at some point in the future by someone, which would be enough to actuate the first good, the fact that people tend to want to preserve their material for their own use, rather than to donate, suggests that they do not simply want to reproduce, but that they are looking for a parenting experience. That said, we feel it is reasonable to say that the primary aim of FP is to preserve a patient’s ability to experience being a parent to their genetically related child, as opposed to merely being a genetic progenitor in the absence of a parenting experience. Thus this includes the possibility of using surrogacy where, while another person carries the pregnancy, the original patient has both a genetically related child and a parenting experience.

Of course, in the context of an adult patient with a partner, the aim of FP can also be to preserve the opportunity for that partner to have the experience of being a parent to children genetically related to the patient and the surviving partner. Consistent with what we have said above, we contend that the good derived from this is that of enabling the surviving partner to have a parenting relationship in a way that connects them to the deceased patient, rather than the good of simply having genetically reproduced (which could be achieved by donation to a stranger).

Having now established that the purpose of FP is not simply to facilitate genetic reproduction *qua* genetic reproduction but rather to preserve the option of having a parenting experience with one’s genetic progeny for either oneself or one’s partner, we will go on to consider arguments for and against routinely offering FP.

### Respecting autonomy

Respecting autonomy is a cornerstone of Western medical ethics, and this requires us to respect a person’s right to make decisions for themselves, including about their medical care. Respect for autonomy does not mean we must do whatever people want, but that we take their wishes seriously and allow them to choose when a choice is available. In order to act autonomously, people need to be sufficiently informed about what their options are and the likely consequences of decision options – a fundamental prerequisite for ensuring informed consent.

Increasingly, patients (including cancer patients) expect to participate and be involved in their treatment decisions (Brietsameter 2010, Siminoff & Thomson 2010). Shared decision-making is a process whereby clinicians share information about treatment options, empowering the patient to actively decide based on their preferences (Elwyn *et al.* 2012) and this process is considered a quality benchmark for the delivery of dignified care (Coulter & Collins 2011, Department of Health 2012).

As such, in order to respect autonomy, a high-quality fertility discussion in oncology is critical to ensure that cancer patients are sufficiently informed about the potential impact of cancer upon fertility. This will support them to make autonomous, fully informed decisions that give them control over their reproductive future, before giving consent to any subsequent intervention.

Hudson *et al.* (2016) have noted that the need to respect patient's freedom of thought, intention and action while delivering healthcare, seems to suggest that everyone should be offered FP. This seems plausible. An autonomy-based argument can clearly be made for having an FP conversation with every patient or the patient’s proxy decision maker (i.e. a parent) if they are not able to make a decision by virtue of their age, or a best interest decision made in accordance with the Mental Capacity Act (2005) if they are an adult who is otherwise incapacitous. Not to raise the issue of fertility loss, and inform patients of FP options, would be a *de facto* deception by omission and this is an insult to autonomy.

Hudson *et al.* (2016) do sound word of caution about drawing this conclusion, however, asking whether cancer patients with a poor prognosis are really able to make autonomous decisions? For example, the extent to which cancer patients can actually make informed decisions regarding posthumous reproduction PAR has been debated, as a result of the time pressure needed to make an FP decision and emotional and cognitive factors which may inhibit the ability to retain information (Lawson *et al.* 2015). In our view, while it is, of course, possible that some patients in this position will not have the capacity to make a complex future-looking decision about their fertility, capacity is person and decision specific, and no blanket assumption can be made.

That said, although there is a strong *prima facie* argument for respecting autonomy by giving (capacitous) patients the option of FP, various studies suggest that doctors are not providing all patients with information about FP (Meyer & Farrell 2015). Many healthcare professionals report a decreased likelihood of initiating discussions related to FP with patients who have a poor prognosis. Poor prognosis has been cited as a major barrier for talking about FP with patients by 66.9% of oncologists (Zhang *et al.* 2020), with other studies citing that over 50% of oncologists state that a patient’s poor prognosis may dissuade them from discussing FP (Forman *et al.* 2009, Sallem *et al.* 2018). Comparable findings have been reported in other studies, with 41–88% of clinicians citing poor prognosis as a factor that would either influence FP discussions or lead them to not offer the option to patients (Collins *et al.* 2011, Adams *et al.* 2013, Louwé *et al.* 2013, Chung *et al.* 2017). Similarly, a survey of paediatric oncologists’ attitudes and practices towards FP in adolescents reported that a patient having a poor survival prognosis was one of three most likely reasons for the physician to not recommend sperm banking (Köhler *et al.* 2011).

Quinn *et al.* (2009) reported that the majority of oncologists included in their research do not discuss FP with patients with a poor prognosis. One oncologist reported that they experienced discomfort at the thought of talking about ‘future babies’ with a patient that is unlikely to be alive within months. The authors concluded that although guidelines suggest healthcare professionals should discuss FP with all patients, the majority appear to not follow these guidelines. Takeuchi *et al.* (2017) observed an internal conflict in clinicians regarding whether, and when, it was appropriate to discuss FP with patients with a poor prognosis.

This evidence about clinician practice tallies with studies reporting patient experience, with Jones *et al.* (2022) reporting patients saying they were not informed about or offered FP at cancer diagnosis. One explanation for this may be that clinician preferences can be limited by ‘implicit persuasion’. This is a process whereby clinicians subconsciously place greater emphasis on the treatment options they consider to be better suited to the patient, a phenomenon frequently observed in oncology settings (Engelhardt *et al.* 2016). When an oncologist’s goal is to save lives and treat the cancer, they may see that as taking precedence and discussion about FP a distraction that could delay treatment and thus worsen prognosis at the detriment to the patient (Hudson *et al.* 2016). This may be a conscious omission (e.g. due to the pressure to initiate cancer treatment), or an unconscious bias. The former could be either an unethical insult to autonomy or an act of beneficent paternalism, whereas the latter would seem to be a form of negligent or unprincipled omission.

While some clinicians may take a conscious position against offering FP, or simply not think to mention it, a number of studies have reported that many healthcare professionals take a neutral stance or have a mixed responses to fertility issues in cancer patients with a poor prognosis. Quinn *et al.* (2012) reported that 45.2% of oncologists had a neutral stance on the issue of patients with a poor prognosis pursuing FP. When questioned about posthumous parenting, 16.2% of oncologists expressed their support for this, while the majority (51.5%) did not have an opinion on the matter. Oncologists who were aware of the American Society of Clinical Oncology guidelines (which highlight the importance of patient education and acknowledge the need for oncologists to provide fertility-related information) were more likely to discuss fertility issues with patients with a poor prognosis. Rosenberg *et al.* (2017) found that 58% of oncologists believed that patients with a poor prognosis should not pursue FP. However, overall, this belief did not appear to dissuade the majority of oncologists from discussing fertility issues with this patient group. When asked whether they discuss fertility issues with this patient group, 84% of oncologists reported that they either always or usually had these discussions (43% and 41%, respectively), while 15% rarely did and 1% never had these discussions.

Some studies suggest that nurses may be more inclined to discuss FP with all patients, regardless of prognosis. In a study by Vadaparampil (2007), paediatric oncology nurses were asked about discussing FP options with patients. The majority (68%) of nurses stated that a patient’s poor prognosis would not affect the discussion being carried out. Just over one quarter (28%) of nurses stated that they would be less likely to discuss FP options with this group of patients, and 4% stated that a patient’s poor prognosis would make them more likely to discuss FP options. Comparable findings were observed by Krouwel *et al.* (2017), who carried out a survey study with 421 oncology nurses to investigate their knowledge about FP and possible barriers to discussions of this topic. When asked to rate their agreement with the statement: *‘*I would tend not to discuss FP with a patient because the patient has poor prognosis’, over half of nurses (55.2%) strongly disagreed with this statement. Over a quarter (28.4%) of participants stated that they neither agreed nor disagreed with this statement, with 16.4% strongly agreeing with the statement. Similarly, in a study comparing nurses’ views on FP in patients with a poor prognosis between 2006 and 2005 (Clayton *et al.* 2008), regardless of the survey year, the majority of nurses reported that a poor prognosis would not affect the likelihood of FP discussions. The number of nurses reporting that FP discussions were more likely for patients with a poor prognosis also increased from 2005 (5%) to 2006 (22%), in addition to a slight decrease in the number of nurses reporting that they would be less likely to discuss FP with patients with a poor prognosis (this figure was 27% in 2006 and 28% in 2005). However, King *et al.* (2008) reported that the odds of a patient surviving was a factor that determined whether nurses discussed FP with their patients.

What we can take from this is that there is mixed practice, but whereas the clinicians who always offer FP will always be respecting autonomy, those who never or only sometimes do seem to be acting, at least *prima facie*, unethically, in light of the strong autonomy argument outlined above. The question, then, is whether these data evidence an ethical failure on the part of many clinicians, or whether this variation in practice should give us a pause to ask whether there are valid reasons that override the *prima facie* autonomy argument. It is the latter possibility that we will now explore.

### Justice and opportunity cost

The argument of justice and opportunity cost is concerned with resource prioritisation, and might contend that FP, in cases where it will not lead to a parenting experience, is futile and therefore not a good use of resource. Crystalising this more generally for a publicly funded health system, where treatment prioritisation decisions will be based on cost-effectiveness metrics, this is because the decision to fund FP where the prognosis is poor cannot be justified at a societal level as a reasonable distribution of scare resources. It can also plausibly be argued that having genetically related children, while very important to many people, is not a need but rather a desire (McTernan 2015). Even without taking a position on that, however, it seems that FP cannot achieve its aims unless it has a chance of resulting in a parenting experience - which it does not if the prognosis is very poor, and the patient is unlikely to survive to be a parent. This argument would, then, suggest that FP should not be offered to patients where FP will not result in a meaningful parenting experience for either them or their partner – and so would rule out FP for children and single adults who are unlikely to survive to become parents.

This perhaps gives us reason to revisit our previous claims about the purpose of FP. If we are going to rule out FP for some patients on the basis of futility, we ought to be sure that there are not other reasons that might justify providing it, even when a parenting experience cannot be achieved.

### Hope and imagined futures

There are two linked arguments to consider here, one centred on FP providing hope for a future and the other on an imagined future as a parent.

### Hope for a future

It is well evidenced that the experience of infertility can lead to distress which can influence the individual emotionally in the short term, and also their sense of identity and expectations for the future (Letherby 1999). Franklin (2022) has suggested that IVF and its related technologies are ‘hope technologies’, because they offer an enticing technological solution to the enigmatic problem of infertility, and in this context, clinicians become providers of hope. It can be argued that for cancer patients with a poor prognosis, FP treatments, which will often involve assisted reproduction techniques, are also hope technologies. Hope has also been conceptualised and operationalised by Snyder (1995), who contends that its existence is essential as a psychological coping strategy, and it could be argued that everything that can be done to better support poor prognosis patients at this time should be done. However, cognitive rules which govern the appropriateness of hope include such criteria as the goal being under some control and at the mid-range in terms of its probability of being attained (Averill *et al.* 2012).

When the prognosis is good, it is not contentious to assume that offering FP treatment is appropriate because it is nurturing realistic hope of future parenthood. In contrast, where patients have a poor prognosis, where even the probability of using the stored material is low (yet alone success rates from any subsequent IVF/surgery), it may be deemed that nurturing hope by offering procedures, in these circumstances, is considered inappropriate. Furthermore, the act of offering FP to a patient with a very poor prognosis might itself offer unrealistic hope of survival, and may be interpreted as saying that there is good chance of living to become a parent – because why offer it otherwise? Indeed,Vadaparampil *et al.* (2008) found in their qualitative study that many of the 24 paediatric oncologists described how just mentioning the need for FP was seen as a sign of hope for patients and families.

The offer of FP, then, essentially becomes an offer of hope for a future which is false hope. It may be that in some cases hope, false or not, can provide benefit, and so becomes a benevolent lie – but in other cases giving false hope will be harmful, leading to a loss of trust and increased trauma when the falsehood becomes apparent. The potential harm arising from false hope exposed might be amplified if the retrieval intervention was burdensome to the patient, and the wrong amplified if it was costly to the health service or patient (if self-funded).

As such, we feel that offering FP to provide false hope as a therapeutic intervention is highly problematic, akin to a lie. Such a benevolent lie might, on occasion, be justified but would be the exception rather than the norm. However, it may be possible for a patient to benefit from an imagined future as a parent, even in the knowledge that they will never become one.

There is one further important argument to make here. It could be argued that the speed of medical progress is high; so that what is an incurable cancer today may be treatable tomorrow. Furthermore, clinical judgement is fallible and patients who are believed to be terminal may not be. This could lead to the argument that we should offer FP treatment to everyone because (a) the terminal diagnosis may be wrong, and (b) the currently terminally ill patients may end up surviving due to advances in medical treatments. In both cases, the surviving patients will lose their ability to reproduce if not offered FP. Therefore, not offering FP to a patient due to their poor prognosis may not be justified. Some patients may survive and deeply regret their lost fertility, while those who do not survive and have their fertility preserved are no worse off.

This argument has some merit, but it is grounded in the assumption that it is preferable to provide a costly service to ensure that no one might miss an opportunity to parent, and it is not clear that this is a proportionate or justifiable response, especially when the potential benefit of a live birth is not guaranteed. The possibility of diagnostic error, or the possibility that cure may be around the corner, is not, in our opinion, enough to offset entirely the problems we have outlined. Rather, the possibility and likelihood of both would (and we suggest usually would) be routinely factored into prognostication and therefore into the decision about whether to offer FP.

### Imagined future as a parent

One reason sometimes cited for pursuing FP when diagnosed with a cancer with a poor prognosis is the desire for a ‘genetic legacy’ (Hudson *et al.* 2016). As mentioned above, FP can result in the production of an offspring that can continue the cancer patient’s bloodline through the process of posthumous reproduction (Quinn *et al.* 2012). There is some evidence to suggest that the public view PAR favourably (Barton *et al.* 2012, Hans & Dooley 2014), although PAR may be viewed negatively by oncologists when it comes to teenage and young adult and paediatric cancer patients (Quinn & Vadaparampil 2011) and a therefore a barrier to an FP discussion.

The argument of imagined futures is different to an argument of genetic legacy. As mentioned above, there is reason to be sceptical about a genetic legacy motivation purely because people exploring FP do not tend to do so with the aim of donating their material for just anyone to reproduce with (which would satisfy the need for genetic legacy), but for either their own use or the use of someone they already have a relationship with. The argument of imagined futures, instead, is based on the good that might come from the dying cancer patient, who knows they will never have a parenting experience, taking comfort in imagining their future child. For those patients where future parenthood had always been part of their sense of identity and long-term goals, the comfort gained from taking concrete steps that allow them to vividly imagine the existence of their child, and a parent–child relationship, may in itself be enough to justify offering FP even to patients who know and understand they will certainly not survive to parent.

This imagining might also include imagining a future in which their partner and child are flourishing and getting comfort from each other, which leads to the argument of gifting.

### Gifting

Here, a cancer patient might see FP as a way to gift the opportunity to become a parent to the partner who will survive them, and with whom they may have planned to have children. This may be linked to the argument of imagined futures, but it need not be. It may simply be an act of generosity, where they wish to gift something to someone they love, even though they will never see or experience the result – and there seems little to object to ethically in such an act.

Something that ought to be considered in light of both of these arguments, however, is the pressure that cancer patients may feel under to act as though they value reproduction and engage with FP simply to please others because they feel it is expected of them. We live in a pro-natalist society, where reproduction is the norm (Greil *et al.* 2011), and in which people are more often required to explain their decision not to have children than their decision to have them. Indeed, the act of simply offering FP is enough to suggest that it is important to preserve fertility. In this context, where people may feel under pressure to ‘gift’ their reproductive tissue or act as if the thought of having children brings them comfort, it is important that if and when FP is raised, it is presented as a genuinely neutral choice.

So far, we have considered the argument of autonomy, which gives us good reason to offer FP to everyone, followed by the argument of justice and opportunity cost that suggests it would be justifiable to not offer FP to patients with poor prognosis because they would never experience the goods of being a parent. We then considered two arguments, the argument of imagined futures (including hope) and the argument of gifting, which support the idea that patients may, nonetheless, wish to consider FP for reasons other than having a parenting experience themselves. These latter two arguments do, however, appear to limit the scope of to whom FP should be offered, to include cancer patients who have a partner to whom they wish to give the gift of becoming a parent, or anyone who would be comforted by imagining a relationship with their future children (even if they will not experience it). This would seem to exclude, however, in all cases, child patients, who would not have a partner to gift to and seem very unlikely to be able to imagine a parental relationship and gain comfort from it.

This exclusion of children with poor prognosis from FP might be supported by other arguments, notably the need to protect them from undergoing interventions that will not benefit them, and which they might consent to undertake in order to please others, because they think they ought to do it (see above comment about pro-natalism).

Essentially, there are good reasons to think that FP can and should be offered to patients with poor prognosis, but only when they have the capacity to benefit in some way. In the absence of that capacity to benefit, there is little justification for offering FP and indeed given the risk of the offer itself being pressuring there are prima facie reasons not to offer it.

So far, the arguments we have considered have been patient focussed, and it is worth briefly considering some wider arguments that could impact on the summary conclusion outlined above.

### The welfare of the subsequent child

Arguments based on the welfare of the child are sometimes made to either support or oppose FP in this context. We do not find any of them convincing and so will outline them only briefly for completeness.

First, it might be argued that FP should not be offered to any patient with a poor prognosis because any resultant child will be harmed by not having one of its parents. This argument can be expressed in different ways. Hudson *et al.* (2016) and Quinn *et al.* (2012) note that there could be economic and social harms to a child raised without one parent. Taking a different line, Lawson *et al.* (2016) have suggested children conceived via posthumous reproduction can be compared to ‘replacement children’, a term used by researchers to describe children who are born following the death of an older sibling and may therefore be placed under unreasonable pressure to be a good replacement.

We would resist these arguments. First, they speculate about necessary harm to children raised in one parent families, which is not corroborated by evidence. Studies have highlighted that children raised to single mothers through donor insemination display fewer emotional and behavioural difficulties than those raised by married couples who have used donor insemination (Murray & Golombok 2005), in addition to solo women reporting higher education levels, higher income professions, and equally or higher perceived social support from friends and family in comparison to cohabiting women awaiting fertility treatment (Lindell Pettersson *et al.* 2023). Second, it problematically assumes that the surviving partner would never find another partner who would be a step-parent or that they will themselves be an unfit parent. Third, it assumes that a life with only one parent is not only sub-optimal but is a life not worth living – which seems very problematic and would be contested by many people so raised. A longitudinal study exploring changing family dynamics in over 27,800 single parent households reported no evidence of a negative impact from living in a one parent household on children’s well-being, with children reporting equally high, or higher, scores in various measures of well-being compared to those who have always lived in a two-parent household (Rabindrakumar 2018). Furthermore, if there is genuine concern about child welfare linked to financial and home situations, then there should be financial and welfare thresholds for all people having children – not only where assistance is needed. We therefore dismiss this concern and note that the ASRM guidelines state that concerns about the welfare of the offspring are not sufficient cause for denying FP (The Ethics Committee of the American Society for Reproductive Medicine 2005).

Second, it might be argued that by not offering FP, possible children are harmed by not being brought into existence. We raise this only to dismiss it quickly, as it does not seem feasible that we could consider harm being affected to those who are never brought into existence, as there is no subject that could be harmed.

### The welfare of the partner

Tremellen and Savulescu (2015) note that, in the context of posthumous reproduction, there could be implications for the welfare and quality of life of the partner, specifically in terms of raising a child in grief. Hudson *et al.* (2016) also consider this, noting that

...research is needed to explore thelong-term impact on widowed partners and their offspring. Partners in this precarious position shouldreceive counselling and support during this decision-making process.

There are also the general and ubiquitous challenges, both financial and emotional issues of single parenting. One immediate response is to note that a surviving partner is under no obligation to use the preserved material. But it is plausible to think that they may feel under pressure to use it because it is there. There may also be a financial cost of continuing storage. That said, this does not, in our view, provide reason to not offer FP at all but rather (as noted by Hudson *et al.* 2016) to ensure that a surviving partner is appropriately supported, and enters into, or chooses not to enter into, a posthumous parenting project autonomously.

### Concluding argument

In the discussion above we have considered a range of arguments both for and against routinely offering fertility preservation to patients with poor prognosis. It is clear that there is a strong *pro* *tanto* argument, grounded in respect for autonomy, that supports routinely offering to all patients. We have, however, also shown that there are good reasons to sometimes be cautious about making this offer, not least because there are some situations in which fertility preservation would be futile and as such cannot be considered a good use of resource.

The way forward is not absolutely clear; however, we propose that we should adopt a defeasible assumption in favour of offering fertility preservation to all patients who might benefit from it, with the burden of proof on the clinician to show that there are good grounds for withholding the offer. Given that the main argument in favour of routinely offering FP is found in a patient’s autonomous choice to benefit from the intervention, the most appropriate ground for withholding the offer (outside of lack of resource) is if a patient cannot benefit from the intervention.

We must, however, recognise that there are many ways a patient could benefit from fertility preservation, and these are not limited to having a parenting experience. Becoming a parent is arguably the primary goal of fertility preservation, and is one clear benefit, but a patient may also benefit from FP therapeutically in the form of gaining significant comfort from an imagined future, or knowing they have gifted something precious to their partner. That said, the criteria for withholding the offer would hold if and only if FP could not achieve the benefit that is sought. This would make FP a futile treatment and therefore one which there is no obligation to offer.

The challenge here, of course, is that a decision about futility that rests on a clinician’s understanding of the patient’s ability to benefit assumes that a clinician is able to correctly identify all the benefits that a patient might seek to achieve (requiring an intimate and accurate knowledge of the patient and of their partner where present), and accurately predict that these benefits cannot, or are very unlikely to, be achieved. The former would normally be difficult to do without having had a specific conversation with the patient about FP and the reasons why they might want it, and the latter would require a great deal of prognostic certainty.

For this reason, we suggest that the presumption in favour of having a conversation with all patients, including those with very poor prognosis is strong, and can and should only be defeated when there is clear evidence, agreed by a multidisciplinary team, that no benefit is possible. In practice, this might describe a relatively rare situation. One obvious example might be a young child with a negligible to zero chance of survival, who lacks the capacity to benefit from an imagined future, and who is not in a position to want to make a gift to a partner. This describes a patient who cannot themselves benefit from FP, and therefore should not be offered it. This would hold true regardless of whether the child's parents wanted FP, because we assume – we think reasonably – that the justification for fertility treatment lies in the benefit to the patient and not to others, and a child in this position should not be used as a means to achieve a good for their parents. (Note that in the gifting argument FP is justified by the benefit to the patient of giving the gift of parenting to their surviving partner, not by the benefit that may accrue to the surviving partner – although they will certainly benefit as well.)

Given that we are making an argument in favour of a strong presumption of offering FP, one might ask why we do not go so far as to make an argument for it being obligatory. Such an argument might be grounded both in respect for autonomy and a legitimate concern that allowing clinicians any discretion in whether to offer the treatment opens up the possibility of inconsistency and a postcode lottery, where access to treatment depends on the particular views of the clinician in charge. The best way to combat this would be to ensure that every patient, regardless of prognosis, is given equal access. The reason we do not adopt this position is simply that we are more concerned with equity then equality. Nedha (2011, as cited in Paul 2019) defines equality as treating each person the same, regardless of needs and requirements (e.g. in this scenario, providing all patients with the option of FP, regardless of prognosis), whereas equity can be described as treating each individual fairly depending on their needs. Removing clinical discretion and making the offer of FP mandatory in all cases, would ensure every patient is treated equally at a cost of the harms that can follow when the treatment offers false hope, leads to a foreseeable waste of resource, or risks a vulnerable patient feeling pressured into undertaking procedures from which they cannot benefit.

Our position does potentially place a great deal of power in the hands of the clinician as gatekeeper, but this is mitigated by our requirement that the offer of treatment is a defeasible presumption with the requirement of the multi-disciplinary team (MDT) agreement if it is withheld. Placing the burden of proof on the clinical team to show, and agree, that there is no possible benefit to the patient protects the autonomous patient’s right to make their own choices in very large part, while permitting discretion in a few cases where it might be necessary and correct. In adopting this position, we concur with Hudson *et al.* (2016) regarding the need to provide cancer and fertility healthcare professionals with appropriate resources and training packages for addressing the ethical and decision-making implications that arise in these scenarios, and to ensure that informed consent processes are high quality and robust. For those patients who wish to proceed, ongoing care should include appropriate counselling in relation to the possibility, or even probability, of posthumous reproduction. It must be assumed that most patients in this position will not survive, and their preferences for using their frozen gametes or embryos must be clearly documented in line with relevant legislation and local regulations.

In outlining our arguments, we try to find an appropriate balance between ensuring equity of opportunity to beneficial treatment, while allowing for the fact that in some cases FP will be a waste of resource for no patient benefit.

## Declaration of interest

GLJ, AMJ, BP and RAA declare that there is no conflict of interest that could be perceived as prejudicing the impartiality of the opinions reported. JI is member of the NICE Highly Specialised Technology Evaluation Panel. He has not been involved in any NICE discussions involving fertility or fertility preservation, and the views expressed and endorsed here are his and do not represent NICE.

## Funding

This research did not receive any specific grant from any funding agency in the public, commercial or not-for-profit sector. RAA’s work in this field is supported by MRC grant MR/W019140/1.

## Author contribution statement

GLJ conceived the idea for the manuscript, preparing the initial draft. GLJ, AMF and JI critically developed this further, producing the first version, with additional contributions from BP and RAA. All authors edited, reviewed and approved the final manuscript.

## References

[bib1] Adams E Hill E & Watson E 2013 Fertility preservation in cancer survivors: a national survey of oncologists’ current knowledge, practice and attitudes. British Journal of Cancer 108 1602–1615. (10.1038/bjc.2013.139) 23579214 PMC3668471

[bib2] Averill JR Catlin G & Chon KK 2012 Rules of Hope. New York: Springer.

[bib3] Barton SE Correia KF Shalev S Missmer SA Lehmann LS Shah DK & Ginsburg ES 2012 Population-based study of attitudes toward posthumous reproduction. Fertility and Sterility 98 735–740.e5. (10.1016/j.fertnstert.2012.05.044) 22763100

[bib4] Breitsameter C 2010 Medical decision-making and communication of risks: an ethical perspective. Journal of Medical Ethics 36 349–352. (10.1136/jme.2009.033282) 20511352

[bib5] Chung JP Lao TT & Li TC 2017 Evaluation of the awareness of, attitude to, and knowledge about fertility preservation in cancer patients among clinical practitioners in Hong Kong. Hong Kong Medical Journal 23 556–561. (10.12809/hkmj176840) 29123075

[bib6] Clayton H Quinn GP Lee JH King LM Miree CA Nieder M & Vadaparampil ST 2008 Trends in clinical practice and nurses’ attitudes about fertility preservation for pediatric patients with cancer. Oncology Nursing Forum 35 249–255. (10.1188/08.ONF.249-255) 18321837

[bib7] Collins IM Fay L & Kennedy MJ 2011 Strategies for fertility preservation after chemotherapy: awareness among Irish cancer specialists. Irish Medical Journal 104 6–9. 21391329

[bib8] Coulter A & Collins A 2011 Making Shared Decision Making a Reality: No Decision About Me Without Me.: London: The King’s Fund.

[bib9] **Department of Health (DOH)** 2012 Liberating the NHS: no decision about me without Me. Available at: https://assets.publishing.service.gov.uk/government/uploads/system/uploads/attachment_data/file/216980/Liberating-the-NHS-No-decision-about-me-without-me-Government-response.pdf (Accessed 6 June 2023)

[bib10] Elliot MK 2004 Tales of parenthood from the Crypt: the predicament of the posthumously conceived child. Real Property, Probate and Trust Journal 39 47–69.

[bib11] Elwyn G Frosch D Thomson R Joseph-Williams N Lloyd A Kinnersley P Cording E Tomson D Dodd C Rollnick S, 2012 Shared decision making: a model for clinical practice. Journal of General Internal Medicine 27 1361–1367. (10.1007/s11606-012-2077-6) 22618581 PMC3445676

[bib12] Engelhardt EG Pieterse AH van der Hout A de Haes HJ Kroep JR van Ufford-Mannesse PQ Portielje JE Smets EM & Stiggelbout AM 2016 Use of implicit persuasion in decision making about adjuvant cancer treatment: a potential barrier to shared decision making. European Journal of Cancer 66 55–66. (10.1016/j.ejca.2016.07.011) 27525573

[bib13] **ESHRE** G uideline of the European S ociety for Human Reproduction and Embryology Female Fertility Preservation 2020. Available at: https://www.eshre.eu/Guidelines-and-Legal/Guidelines/Female-fertility-preservation Accessed 13 June 2023.

[bib14] Forman EJ Anders CK & Behera MA 2009 Pilot survey of oncologists regarding treatment-related infertility and fertility preservation in female cancer patients. Journal of Reproductive Medicine 54 203–207. 19438160 PMC2903537

[bib15] Franklin S 2022 Embodied Progress: A Cultural Account of Assisted Conception. Oxon: Taylor & Francis.

[bib16] Greil A McQuillan J & Slauson‐Blevins K 2011 The social construction of infertility. Sociology Compass 5 736–746. (10.1111/j.1751-9020.2011.00397.x)

[bib17] Hans JD & Dooley B 2014 Attitudes toward making babies … with a deceased Partner’s cryopreserved gametes. Death Studies 38 571–581. (10.1080/07481187.2013.809033) 25010854

[bib18] Hudson J Vadaparampil ST Tamargo C & Quinn GP 2016 Fertility preservation in cancer patients with a poor prognosis: the controversy of posthumous reproduction. Future Oncology 12 1675–1677. (10.2217/fon.15.270) 26984472

[bib19] Jones GL Moss RH Darby F Mahmoodi N Phillips B Hughes J Vogt KS Greenfield DM Brauten-Smith G Gath J, 2022 Cancer, Fertility and Me: developing and testing a novel fertility preservation patient decision aid to support women at risk of losing their fertility because of cancer treatment. Frontiers in Oncology 12 896939. (10.3389/fonc.2022.896939) 35847858 PMC9280471

[bib20] King L Quinn GP Vadaparampil ST Gwede CK Miree CA Wilson C Clayton H & Perrin K 2008 Oncology nurses’ perceptions of barriers to discussion of fertility preservation with patients with cancer. Clinical Journal of Oncology Nursing 12 467–476. (10.1188/08.CJON.467-476) 18515245

[bib21] Köhler TS Kondapalli LA Shah A Chan S Woodruff TK & Brannigan RE 2011 Results from the survey for preservation of adolescent reproduction (SPARE) study: gender disparity in delivery of fertility preservation message to adolescents with cancer. Journal of Assisted Reproduction and Genetics 28 269–277. (10.1007/s10815-010-9504-6) 21110080 PMC3082660

[bib22] Krouwel EM Nicolai MPJ van Steijn-van Tol AQMJ Putter H Osanto S Pelger RCM & Elzevier HW 2017 Fertility preservation counselling in Dutch Oncology Practice: are nurses ready to assist physicians? European Journal of Cancer Care 26 e12614. (10.1111/ecc.12614) 28026055

[bib23] Lambertini M Del Mastro L Pescio MC Andersen CY Azim HA Peccatori FA Costa M Revelli A Salvagno F Gennari A, 2016 Cancer and fertility preservation: international recommendations from an expert meeting. BMC Medicine 14 1. (10.1186/s12916-015-0545-7) 26728489 PMC4700580

[bib24] Lambertini M Peccatori FA Demeestere I Amant F Wyns C Stukenborg JB Paluch-Shimon S Halaska MJ Uzan C Meissner J, 2020 Fertility preservation and post-treatment pregnancies in post-pubertal cancer patients: ESMO Clinical Practice Guidelines†. Annals of Oncology 31 1664–1678. (10.1016/j.annonc.2020.09.006) 32976936

[bib25] Lawson AK Klock SC Pavone ME Hirshfeld-Cytron J Smith KN & Kazer RR 2015 Psychological counseling of female fertility preservation patients. Journal of Psychosocial Oncology 33 333–353. (10.1080/07347332.2015.1045677) 25996581 PMC4557874

[bib26] Lawson AK Zweifel JE & Klock SC 2016 Blurring the line between life and death: a review of the psychological and ethical concerns related to posthumous-assisted reproduction. European Journal of Contraception and Reproductive Health Care 21 339–346. (10.1080/13625187.2016.1203892) 27388465

[bib27] Lee SJ Schover LR Partridge AH Patrizio P Wallace WH Hagerty K Beck LN Brennan LV Oktay K & American Society of Clinical Oncology 2006 American Society of Clinical Oncology recommendations on fertility preservation in cancer patients. Journal of Clinical Oncology 24 2917–2931. (10.1200/JCO.2006.06.5888) 16651642

[bib28] Letherby G 1999 Other than mother and mothers as others. Women’s Studies International Forum 22 359–372. (10.1016/S0277-5395(9900028-X)

[bib29] Lindell Pettersson M Sydsjö G Lampic C Skoog Svanberg A & Elenis E 2023 Perceived social support in solo women seeking treatment with donor gametes and in women in heterosexual couples seeking IVF-treatment with own gametes. Scientific Reports 13 2733. (10.1038/s41598-023-29441-y) 36792663 PMC9931690

[bib30] Loren AW Mangu PB Beck LN Brennan L Magdalinski AJ Partridge AH Quinn G Wallace WH Oktay K & American Society of Clinical Oncology 2013 Fertility preservation for patients with cancer: American Society of Clinical Oncology clinical practice guideline update. Journal of Clinical Oncology 31 2500–2510. (10.1200/JCO.2013.49.2678) 23715580 PMC5321083

[bib31] Louwé LA ter Kuile MM Hilders CGJM Jenninga E Tiemessen SM Peters AA Nortier JW & Stiggelbout AM 2013 Oncologists’ practice and attitudes regarding fertility preservation in female cancer patients: a pilot study in the Netherlands. Journal of Psychosomatic Obstetrics and Gynaecology 34 129–132. (10.3109/0167482X.2013.821977) 23915206

[bib32] McTernan E 2015 Should fertility treatment be state funded? Journal of Applied Philosophy 32 227–240. (10.1111/japp.12091)

[bib33] Meyer F & Farrell E 2015 Ethical dilemmas in palliative care: a case study of fertility preservation in the context of metastatic cancer. Journal of Palliative Medicine 18 661. (10.1089/jpm.2015.0169) 26098359

[bib34] Muñoz M Santaballa A Seguí MA Beato C De La Cruz S Espinosa J Fonseca PJ Perez J Quintanar T & Blasco A 2016 SEOM Clinical Guideline of fertility preservation and reproduction in cancer patients (2016). Clinical and Translational Oncology 18 1229–1236. (10.1007/s12094-016-1587-9) 27896641 PMC5138251

[bib35] Murray C & Golombok S 2005 Solo mothers and their donor insemination infants: follow-up at age 2 years. Human Reproduction 20 1655–1660. (10.1093/humrep/deh823) 15734751

[bib36] **National Institute for Health and Care** **Excellence (NICE)** Cryopreservation to preserve fertility in people diagnosed with cancer. Available at: http://pathways.nice.org.uk/pathways/fertility#path=view%3A/pathways/fertility/cryopreservation-to-preserve-fertility-in-people-diagnosed-withcancer.xml&content=view-index (Accessed 13 June 2023)

[bib37] Paul HJ 2019 Equity vs equality: facilitating equity in the classroom. International Journal of Research and Scientific Innovation 6 216–219.

[bib38] Peate M Meiser B Hickey M & Friedlander M 2009 The fertility-related concerns, needs and preferences of younger women with breast cancer: a systematic review. Breast Cancer Research and Treatment 116 215–223. (10.1007/s10549-009-0401-6) 19390962

[bib39] Peccatori FA Azim HA Orecchia R Hoekstra HJ Pavlidis N Kesic V & Pentheroudakis G 2013 Cancer, pregnancy and fertility: ESMO Clinical Practice Guidelines for diagnosis, treatment and follow-up. Annals of Oncology 24(6) 160–170. (10.1093/annonc/mdt199) 23813932

[bib41] Quinn GP & Vadaparampil ST 2011 Fertility and cancer – patient, physician, and institutional barriers. US. Oncology and Hematology 7 22–24. (10.17925/OHR.2011.07.1.22)

[bib40] Quinn GP Vadaparampil ST King L Miree CA Wilson C Raj O Watson J Lopez A & Albrecht TL 2009 Impact of physicians’ personal discomfort and patient prognosis on discussion of fertility preservation with young cancer patients. Patient Education and Counseling 77 338–343. (10.1016/j.pec.2009.09.007) 19796912

[bib42] Quinn GP Knapp CA Malo TL McIntyre J Jacobsen PB & Vadaparampil ST 2012 Physicians’ undecided attitudes toward posthumous reproduction: fertility preservation in cancer patients with a poor prognosis. Journal of Supportive Oncology 10 160–165. (10.1016/j.suponc.2011.09.006) 22266153 PMC4533919

[bib43] Rabindrakumar S 2018 Family Portrait: single parent families and transitions over time. Available at: https://www.greenme.com.br/wpcontent/uploads/2019/01/Sheffield_Solutions_Modern_Families.pdf (Accessed 8 June 2023)

[bib44] Rosenberg SM Gelber S Gelber RD Krop E Korde LA Pagani O & Partridge AH 2017 Oncology physicians’ perspectives on practices and barriers to fertility preservation and the feasibility of a prospective study of pregnancy after breast cancer. Journal of Adolescent and Young Adult Oncology 6 429–434. (10.1089/jayao.2017.0031) 28686476 PMC5649410

[bib45] Sallem A Shore J Ray-Coquard I Ferreux L Bourdon M Maignien C Patrat C Wolf JP & Pocate-Cheriet K 2018 Fertility preservation in women with cancer: a national study about French oncologists awareness, experience, and feelings. Journal of Assisted Reproduction and Genetics 35 1843–1850. (10.1007/s10815-018-1251-0) 29974370 PMC6150902

[bib46] Siminoff LA & Thomson MD 2010 The ethics of communication in cancer and palliative care. In Handbook of Communication in Oncology and Palliative Care. New York, NY: Oxford University Press.

[bib47] Snyder CR 1995 Conceptualizing, measuring, and nurturing hope. Journal of Counseling and Development 73 355–360. (10.1002/j.1556-6676.1995.tb01764.x)

[bib48] Takeuchi E Kato M Wada S Yoshida S Shimizu C & Miyoshi Y 2017 Physicians’ practice of discussing fertility preservation with cancer patients and the associated attitudes and barriers. Supportive Care in Cancer 25 1079–1085. (10.1007/s00520-016-3495-5) 27889828

[bib49] **The Ethics Committee of the American Society for Reproductive Medicine** 2005 Fertility preservation and reproduction in cancer patients. Fertility and Sterility 83 1622–1628. (10.1016/j.fertnstert.2005.03.013) 15950628

[bib50] Tremellen K & Savulescu J 2015 A discussion supporting presumed consent for posthumous sperm procurement and conception. Reproductive Biomedicine Online 30 6–13. (10.1016/j.rbmo.2014.10.001) 25456161

[bib51] Vadaparampil ST Clayton H Quinn GP King LM Nieder M & Wilson C 2007 Pediatric oncology nurses’ attitudes related to discussing fertility preservation with pediatric cancer patients and their families. Journal of Pediatric Oncology Nursing 24 255–263. (10.1177/1043454207303878) 17827491

[bib52] Vadaparampil S Quinn G King L Wilson C & Nieder M 2008 Barriers to fertility preservation among pediatric oncologists. Patient Education and Counseling 72 402–410. (10.1016/j.pec.2008.05.013) 18621502

[bib53] Yasmin E Balachandren N Davies MC Jones GL Lane S Mathur R Webber L Anderson RA & British Fertility Society 2018 Fertility preservation for medical reasons in girls and women: British fertility society policy and practice guideline. Human Fertility 21 3–26. (10.1080/14647273.2017.1422297) 29298394

[bib54] Zhang H Wang G Jiang B Cao M Jiang Q Yin L Fu B & Zhang J 2020 The knowledge, attitude, and self-reported behaviors of oncology physicians regarding fertility preservation in adult cancer patients. Journal of Cancer Education 35 1119–1127. (10.1007/s13187-019-01567-6) 31256354 PMC7679324

